# Quality of Life After Percutaneous Coronary Intervention in No-Touch Saphenous Vein Grafts is Significantly Better Than in Conventional Vein Grafts

**DOI:** 10.21470/1678-9741-2021-0576

**Published:** 2022

**Authors:** Gabriele Ferrari, Jan Karlsson, Yang Cao, Håkan Geijer, Domingos Souza, Ninos Samano

**Affiliations:** 1 Department of Cardiothoracic and Vascular Surgery, Faculty of Medicine and Health, Örebro University, Örebro, Sweden; 2 University Health Care Research Centre, Faculty of Medicine and Health, Örebro University, Örebro, Sweden; 3 Department of Radiology, Faculty of Medicine and Health, Örebro University, Örebro, Sweden; 4 Clinical Epidemiology and Biostatistics, School of Medical Sciences, Örebro University, Örebro, Sweden

**Keywords:** Coronary Artery Bypass, Quality of Life, Percutaneous Coronary Intervention, Saphenous Vein, Fatigue, Propensity Score.

## Abstract

**Objective:**

To compare health-related quality of life (HRQoL) of patients primarily treated with a no-touch saphenous vein graft with that of patients who received a conventional graft.

**Methods:**

The study included all individuals treated with a percutaneous coronary intervention (PCI) on a saphenous vein graft (SVG) between January 2006 and June 2020. The RAND-36 health survey was used to assess HRQoL. The Mann-Whitney U test was used to test differences in HRQoL between the two groups. Effect size was estimated via Cohen’s d. The average treatment effect between the groups was tested by propensity score matching (PSM).

**Results:**

Of the 346 patients treated with a PCI in a stenosed or occluded SVG, 165 responded to RAND-36 (no-touch: n=48; conventional: n=117). Patients with a no-touch graft reported better mean values on seven of the eight health survey domains. Statistically significant differences were observed for four of the domains, all in favour of the no-touch group. The effect size estimates indicated a small difference for five domains, with the highest values (>0.40) seen for the general health and energy/fatigue domains. PSM confirmed a statistically significant difference for the physical functioning and general health domains.

**Conclusion:**

At a mean follow-up of 5.4 years, patients who received a PCI in no-touch vein grafts showed significantly better HRQoL than those who received a PCI in conventional vein grafts.

**Table:** 

Abbreviations, Acronyms & Symbols			
C	= Conventional	NT	= No-touch
CABG	= Coronary artery bypass grafting	P	= Pain
CI	= Confidence interval	PCI	= Percutaneous coronary intervention
EF	= Energy/fatigue	PCS	= Physical component summary
ES	= Effect size	PF	= Physical functioning
EW	= Emotional well-being	PSM	= Propensity score matching
GH	= General health	QoL	= Quality of life
HRQoL	= Health-related quality of life	RE	= Role-functioning/emotional
IQR	= Interquartile range	RP	= Role-functioning/physical
MACE	= Major adverse cardiac events	SD	= Standard deviation
MCID	= Minimal clinically important difference	SF	= Social functioning
MCS	= Mental component summary	SVG	= Saphenous vein graft

## INTRODUCTION

Health-related quality of life (HRQoL) has become an important outcome measure. Most medical treatments are now evaluated not only in terms of clinical/biomarker benefits but also in terms of HRQoL improvements. In 2011, Noyez et al.^[1]^ reviewed the literature regarding HRQoL studies after cardiac surgery. The review showed few HRQoL studies as well as methodological weaknesses such as limited follow-up times and limited sample sizes.

More studies have been published in the last decade, and almost all have used the SF-36 or the RAND-36 to measure HRQoL after cardiac surgery, in particular after coronary artery bypass grafting (CABG) operation^[2^-^11]^. The RAND 36-item health survey 1.0 is a public domain and licence-free form equivalent to the SF-36. The scoring for six of the eight subscales is equivalent for the SF-36 and RAND-36, while scoring for the pain and general health scales differs marginally. RAND-36 is a generic measure of HRQoL that has been validated in the general population and for different patient groups.

To our knowledge, no report published in English has investigated HRQoL in CABG patients who subsequently need a percutaneous coronary intervention (PCI) on a saphenous vein graft (SVG). PCI is an established procedure with excellent results in ischemic heart disease patients, particularly when revascularizing the native coronary arteries^[12]^. On the other hand, PCI of a degenerated SVG often results in a complex percutaneous intervention and its use is debated^[13]^. Controversial results with a high rate of major adverse cardiac events (MACE) have been observed in both the short and long term. No results have yet been reported regarding PCI of a saphenous vein harvested with the no-touch (NT)^[14]^ technique or treated in any other way during the primary CABG operation. The no-touch technique differs from the conventional (C) technique in that it causes less endothelium damage during the harvesting procedure^[15^-^17]^, and leads to reduced neo-intimal hyperplasia and subsequent atherosclerosis in the long term^[18^-^20]^. Our group has previously investigated HRQoL in CABG patients who had received a no-touch vein graft^[11]^, but that study did not compare the no-touch technique with the conventional technique.

The aim of this study was to evaluate HRQoL in individuals who needed a PCI of their SVG after a CABG operation. Our specific aim was to compare HRQoL between patients treated with a no-touch SVG and patients who received a conventional SVG.

## METHODS

### Data Collection

The study cohort consisted of all individuals who underwent a CABG operation at our department between January 1992 and May 2020. The present study included all individuals treated with a PCI on the SVG (stenosed or occluded) between 1 January 2006 and 31 May 2020. The SVG was harvested either with the no-touch technique or with the conventional technique. Two surgeons reviewed all surgical reports to check the categorization of cases into the two groups. The PCI was performed in one of two cardiology departments. The sole exclusion criterion was the execution of PCI less than 30 days after CABG, because this was interpreted as a direct complication of the primary operation due to technical difficulties and not related to the type of vein graft or harvesting technique.

The RAND-36 health survey and information about the study were sent by regular mail to each individual’s address (Supplementary n. 1). In case of non-response, the questionnaire was re-sent at 1-month intervals. All individuals who did not respond the first time were contacted by telephone. Demographic and clinical data were collected from a national quality registry, the Swedish cardiological and cardiosurgical intervention registry (Swedeheart), and from the local hospital register. The study has been approved by the Regional Ethics Review Board in Uppsala (DNR: 2015/242).

**Supplementary 1:**
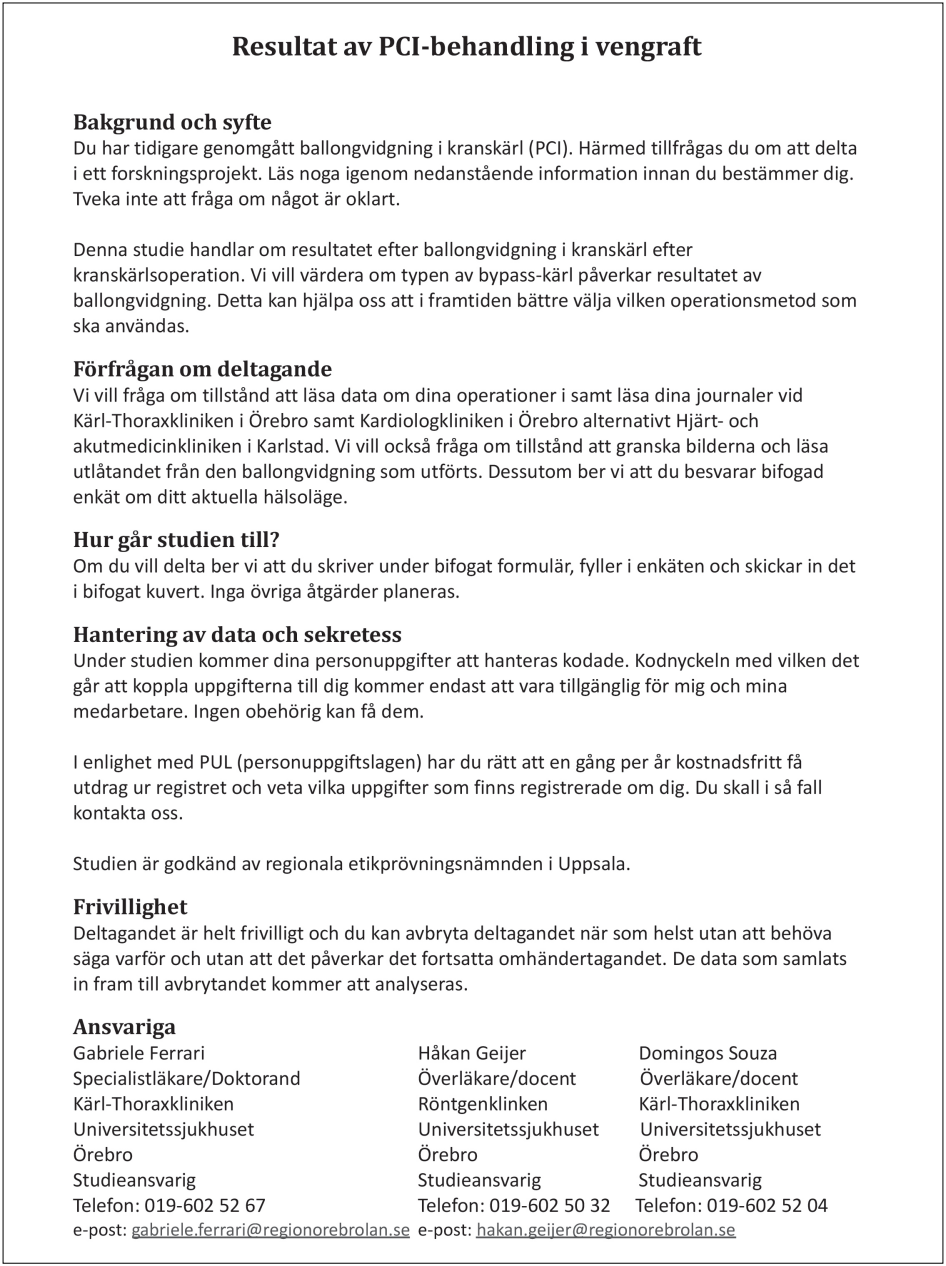
Patient Consensus (in original language).

This study was registered with ClinicalTrials.gov (no. NCT03999398, 25 June 2019) and the Research and Development registry in Sweden (project no: OLL-242381, 17 October 2017).

This was a single-centre study conducted at the centre that invented the no-touch technique and has been using it since 1990. The STROBE checklist for observational studies was followed.

### Patient Cohort

**Fig. 1:**
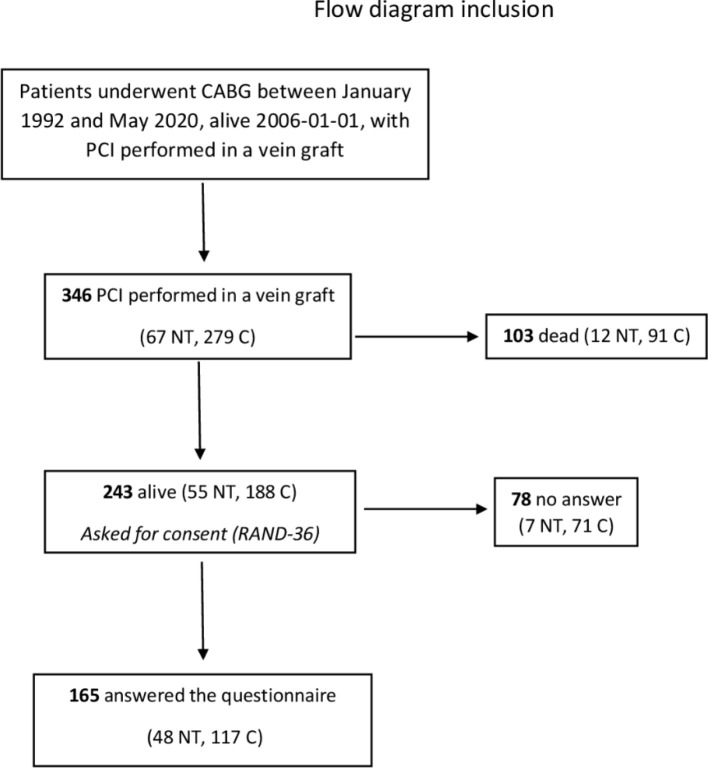
PRISMA flowchart of the individuals included in the study. C=conventional graft; CABG=coronary artery bypass grafting; NT=no-touch graft; PCI=percutaneous coronary intervention.

Between 1 January 2006 and 30 June 2020, 346 patients (67 NT, 279 C) who had previously undergone a CABG surgery were treated with a PCI on the SVG. Of these, 16 patients were excluded because PCI was performed within 30 days of the CABG procedure. A total of 243 patients were alive at the HRQoL follow-up (55 NT and 188 C) and were asked to participate in the study (Figure 1).

### RAND-36 Health Survey

The RAND-36 consists of 36 items grouped into eight multi-item scales: physical functioning (PF), role-functioning/physical (RP), pain (P), general health (GH), energy/fatigue (EF), social functioning (SF), role-functioning/emotional (RE), and emotional well-being (EW). Scale scores are summed and transformed into scales ranging from 0 (worst possible health state) to 100 (best possible state).

### Statistical Analysis

Descriptive statistics were calculated as means, standard deviations (SD), and 95% confidence intervals (CI). Categorical variables were summarized with relative frequency distribution. All continuous data were normally distributed and so summarized with mean and standard deviation (SD). A chi-squared test (or Fisher’s exact test if any expected count was <5) was used to compare categorical values between the two groups. An unpaired t-test was performed to compare continuous variables.

Differences in RAND-36 domains between the two treatment groups were tested with the non-parametric Mann-Whitney U test. The magnitudes of group differences were estimated by calculating the effect size (ES; Cohen’s *d*). ES makes it possible to interpret the importance of a group difference and facilitates comparison across different measures. ES was calculated as the mean difference between groups divided by the pooled SD and was judged according to the standard criteria proposed by Cohen: trivial (0.0 to <0.2), small (0.2 to <0.5), medium (0.5 to <0.8), and large (≥0.8).

In addition, propensity score matching (PSM) was used to estimate the average treatment effects between the two groups, using 1:1 nearest neighbour matching based on the propensity scores. The matched sample size was 96 (48:48). PSM was used as a sensitivity analysis to assess the robustness of the primary analysis results. Propensity scores of patients treated with no-touch or conventional SVG were estimated using a logistic regression model with age, sex, smoking, hypertension, diabetes mellitus, and creatinine level as predictors.

The statistical analyses were performed using SPSS version 27.0 (IBM, Armonk, NY, USA) and Stata version 16.1 (StataCorp, College Station, TX, USA).

## RESULTS

The study included 243 individuals treated with PCI in a stenosed or occluded SVG and alive at the time of the survey. A total of 165 (67.9%) individuals responded to the RAND-36 health survey, 48 (87.3%) in the no-touch group and 117 (62.2%) in the conventional group (Figure 1).

### Demographic and Clinical Characteristics

The demographic and surgical characteristics of the two treatment groups are presented in Tables 1 and 2. The demographic characteristics of the two groups were quite similar, with comparable mean age at time of CABG (57.1±8.6 years *vs.* 57.2±8.1 years, *P*=0.93), at time of PCI (*P*=0.72), and at time of HRQoL follow-up (73.4±8.8 years *vs.* 75.4±7.2 years, *P*=0.12). Most of the patients were male (around 80%). Risk factors were equally distributed in the two groups, and all comparisons of risk factors between the groups were not significant. Regarding the characteristics of the CABG and PCI procedures (Table 2), the two groups showed comparable data in terms of number of anastomoses performed in the CABG, number of successful PCI procedures, frequency of distal embolic protection device usage, and number of thrombectomies performed. The only significant difference was the use of dual antiplatelet therapy at baseline (NT: 54.2% *vs.* C: 72.6%, *P*=0.02). However, after PCI, according to local protocol, the patients received dual antiplatelet therapy for at least 1 year if no contraindication was present.

**Table 1:** Demographic characteristics.

	No-touch	Conventional	*P*-value
Number of patients	48	117	
Age at CABG	57.1±8.6	57.2±8.1	0.935
Age at PCI	70.1±9.1	71.2±8.1	0.725
Age at survey response	73.4±8.8	75.4±7.21	0.124
Time between CABG and PCI	13.6±5.9	14.1±4.8	0.534
Time between PCI and survey	4.4±3.9	5.9±3.5	0.021
Male gender	38 (79.2%)	104 (88.9%)	0.102
Smoking history (past and present)	25 (52.1%)	74 (63.2%)	0.276
Hypertension	43 (89.6%)	98 (83.7%)	0.622
Diabetes mellitus	16 (33.3%)	37 (31.6%)	0.831
Creatinine level	83.8±25.0	90.4±28.3	0.185

Values are presented as mean±standard deviation or n (%). Ages and times are given in years, and creatinine is given in micromole/L. Smoking was divided into three categories in the analysis: never smokers, former smokers (more than 1 month ago), current smokers.

**Table 2:** Surgical and PCI characteristics.

	No-touch	Conventional	*P*-value
Number of patients	48	117	
Number of distal anastomoses	3.7±1.1	3.6±0.9	0.585
Indication for PCI			0.686
Effort angina	18	40	
Acute coronary syndrome	30	77	
Number of stenosed vein grafts	59	170	0.150
PCI success (restenosis <20%)	42 (87.5%)	104 (88.9%)	0.800
Not possible to perform PCI	3 (6.3%)	10 (8.5%)	0.758
Distal embolic protection device	1 (2.1%)	5 (4.3%)	0.673
Thrombectomy performed	6 (12.5%)	9 (7.7%)	0.374
Dual antiplatelet therapy	26 (54.2%)	85 (72.6%)	0.022

Values are presented as mean±standard deviation or n (%). PCI=percutaneous coronary intervention

Analysis of the number of cardiovascular events at 1 year (Table 3) and at long-term follow-up (Table 4) confirmed the comparability of the two groups. At 1 year after PCI, no differences in terms of MACE, in-stent restenosis, or re-hospitalization were reported (Table 3). At the time of HRQoL follow-up (Table 4), more events were reported in the conventional group, but the between-group differences were not statistically significant except for the frequency of in-stent restenosis (NT: 6.25% *vs.* C: 22.2%, *P*=0.01).

**Table 3:** Cardiac events at 1 year.

	No-touch	Conventional	*P*-value
Number of patients	48	117	
Re-angiography during first year			0.706
None	43 (89.6%)	102 (87.2%)	
1	5 (10.4%)	13 (11.1%)	
2	0 (0%)	2 (1.7%)	
Cardiac hospitalization during the 1^st^ year	7 (14.6%)	23 (19.7%)	0.083
In-stent restenosis during the 1^st^ year	0 (0%)	1 (0.8%)	1.000
MACE during the 1^st^ year	7 (14.6%)	23 (19.7%)	0.708

Values are presented as n (%). MACE=major adverse cardiac event

**Table 4:** Cardiac events at health-related quality of life follow-up.

	No-touch	Conventional	*P*-value
Number of patients	48	117	
Re-angiography at follow-up			0.091
None	35 (72.9%)	72 (61.5%)	
1	11 (22.9%)	26 (22.2%)	
2	1 (2.1%)	9 (7.7%)	
3	1 (2.1%)	10 (8.5%)	
In-stent restenosis at follow-up	3 (6.3%)	26 (22.2%)	0.015
Major adverse cardiac event at follow-up	13 (27.1%)	46 (39.3%)	0.072

MACE=major adverse cardiac event

### RAND-36 Health Survey

RAND-36 health profiles divided by type of vein graft are presented in Table 5. The patients treated with a no-touch vein graft reported higher mean values in seven of the eight health domains, indicating better HRQoL. Differences between the two groups were statistically significant (*P*<0.05) in four of the eight domains, all in favour of patients with the no-touch vein graft treatment (*P*=0.028 for physical functioning, *P*=0.022 for general health, *P*=0.010 for energy/fatigue, and *P*=0.026 for emotional well-being). In terms of ES, the between-group differences were trivial (ES <0.20) for the role-functioning/physical, social functioning, and role-functioning/emotional domains, and small (0.20 ≤ ES <0.50) for the other five domains (Table 5). The largest ESs (>0.40) were noted for the general health and energy/fatigue domains.

**Table 5:** Results of the RAND-36 health survey divided by type of vein graft.

RAND-36 domains	No-touch			Conventional			Difference between groups		*P*-value	Effect size
	(n=48)			(n=117)						
	Mean	SD	95% CI	Mean	SD	95% CI	Mean	95% CI		
PF	**68.6**	26.7	62.5-77.9	**58.4**	27.6	53.8-63.9	**10.3**	1.0-19.5	**0.028**	0.38
RP	**47.4**	42.6	36.2-62.6	**40.3**	40.0	32.6-47.6	**7.0**	-6.8-20.1	0.352	0.17
P	**71.8**	25.5	64.2-79.4	**65.0**	29.3	59.9-70.7	**6.8**	-2.9-16.5	0.171	0.24
GH	**61.0**	19.3	55.2-66.7	**52.6**	21.0	49.0-56.8	**8.3**	1.3-15.4	**0.022**	0.41
EF	**65.4**	21.8	58.9-71.9	**55.8**	22.7	51.2-59.9	**9.6**	1.9-17.4	**0.010**	0.43
SF	**77.2**	27.5	69.1-85.4	**72.9**	27.6	68.2-78.4	**4.3**	-5.2-13.8	0.243	0.15
RE	**70.1**	40.2	60.8-84.1	**69.7**	39.5	62.9-77.7	**0.4**	-13.1-13.9	0.851	0.10
EW	**81.0**	21.0	74.8-87.3	**74.9**	20.4	71.3-79.1	**6.1**	-0.9-13.2	**0.026**	0.29

Effect size was calculated according to Cohen’s *d* and categorized as trivial (0.0 to <0.2), small (0.2 to <0.5), medium (0.5 to <0.8), or large (≥0.8). Significant *P*-values are shown in bold. Effect sizes categorized as at least “small” are underlined.

PSM analysis (Table 6) confirmed a statistically significant difference between the two groups for the physical functioning (*P*=0.041) and general health (*P*=0.002) domains. The difference for the energy/fatigue domain showed a borderline trend towards statistical significance (*P*=0.056).

**Table 6:** Treatment effects on the RAND-36 domains of no-touch *versus* conventional vein graft (propensity score matching).

RAND-36 domains	Average treatment effect (95% CI)	*P*-value
PF	**9.31 (0.38-18.24)**	**0.041**
RP	6.03 (-8.27-20.33)	0.409
P	6.83 (-2.92-16.57)	0.170
GH	**10.31 (3.85-16.76)**	**0.002**
EF	8.72 (-0.24-17.68)	0.056
SF	3.86 (-5.73-13.45)	0.430
RE	-0.82 (-15.13-13.49)	0.911
EW	4.62 (-5.78-15.02)	0.384

EF=energy/fatigue; EW=emotional well-being; GH=general health; P=pain; PF=physical functioning; RE=role-functioning/emotional; RP=role-functioning/physical; SF=social functioning

## DISCUSSION

PCI on SVGs has been in continuous evolution over recent decades. It currently represents approximately 6% of all percutaneous coronary procedures in the US^[21]^. Patients undergoing PCI in SVGs have more early and late adverse cardiac events^[22]^, which may predispose them to a deterioration of their HRQoL and increase the burden for healthcare. This is the first study to show a better HRQoL after PCI in no-touch *versus* conventional vein grafts.

Few studies have evaluated HRQoL outcomes after PCI in a vein graft. Our group^[11]^ used the EQ-5D-3L questionnaire to examine HRQoL in individuals after CABG, and concluded that graft patency was associated with better HRQoL. However, the HRQoL outcome of the no-touch or conventional vein graft technique was not evaluated separately.

The present study evaluated HRQoL using the RAND-36 health survey in patients treated with no-touch or conventional vein grafts. Our primary analysis showed significant differences between the two treatment groups in four of the eight RAND-36 domains (PF, GH, EF, EW), indicating better HRQoL in the no-touch group at a mean of 5.4±3.6 years after PCI. The effect size estimates (Cohen’s *d*) indicated better HRQoL in the no-touch group in five domains (PF, P, GH, EF, EW). EFs were small, but the difference in scale scores on the physical functioning and energy/fatigue scales was approximately 10 points, which has been referred to as a mean group difference. The energy/fatigue domain showed the greatest difference (*P*=0.010, ES=0.43). A possible explanation for these results could be the higher in-stent restenosis rate after PCI in the conventional group, and its consequences in terms of quality of life. Further clarifications in terms of clinical outcomes are under investigation with a larger patient cohort (ClinicalTrials.gov no. NCT03999398).

Few studies have estimated the minimal clinically important difference (MCID) for the RAND-36/SF-36 scales in cardiopathic populations. Bjorner et al. evaluated MCID for energy/fatigue in individuals with chronic conditions including congestive heart failure, and recommended a MCID of 5-10 points^[23]^. In the present study, the difference between the no-touch and the conventional groups was 9.6 points for energy/fatigue, indicating a clinically important difference.

The patients who received a no-touch vein graft estimated their physical health (PF, RP, P, GH) more positively than the conventional group, although the differences in RP and P were not significant. The positive effect on physical health in the no-touch group was confirmed by the PSM analysis, showing statistically significant differences in two domains (PF, GH). Comparison of the average treatment effects according to the PSM analysis (Table 6) and differences between treatment groups according to the primary analysis (Table 5) showed that the results were roughly equal. The general similarity between the primary analysis and the PSM indicates that no predictor variable behaved as a confounder in the analysis.

The confirmed positive effect on the physical health components supports the clinical relevance of the HRQoL difference between the two techniques, since the scales that primarily measure physical health are particularly associated with the health condition in cardiac and cardio-operated patients^[24]^.

Hokkanen et al.^[6]^ used RAND-36 to examine both short-term (1 year) and long-term (12 years) changes in HRQoL in patients treated with CABG. Their 1-year results^[25]^ demonstrated that all RAND-36 domains improved significantly; however, this improvement was significant only among patients under 75 years. At the 12-year follow-up, significant improvements were observed in all RAND-36 domains except general health. Moreover, patients younger than 65 years at baseline maintained their physical health status after 12 years, whereas older patients reported a pronounced decrease in both physical and mental component summary scores. The present study was similar in terms of follow-up time, although it was not prospective and did not analyse changes over time. However, an analysis of our results for patients under 65 years showed RAND-36 scores comparable to those reported by Hokkanen et al., particularly in the no-touch group. It is noteworthy that the general health domain did not improve in the earlier study, with a mean value of 54.2 at baseline and 54.5 at 12 years^[6]^. Our study found a significant difference in general health between the no-touch and conventional groups, with a mean value of 61.0 in the no-touch group; the effect size was in the upper range of a small difference (ES=0.41). This result can be explained by the already-known fact that no-touch vein graft patients tend to have reduced atherosclerosis over time and lower rates of adverse cardiac events^[26]^, with an expected positive impact on general health.

Few studies have evaluated HRQoL after PCI using SF-36 or RAND-36^[27^,^28]^. In 2008, Günal et al.^[28]^ reported SF-36 results for octogenarians treated with PCI that partially differ from our results. Their study group showed a markedly lower mean value on the physical functioning scale (41±28) than we found in our study, which may be explained by the demographic characteristics of the study population (patients over 80 years). However, scores on the pain, role-emotional, and emotional well-being scales were comparable with the results for the conventional group in the present study. Cohen et al.^[27]^ investigated HRQoL using the SF-36 after either PCI or CABG at 1 month, 6 months, and 1 year after the intervention, finding no change in HRQoL at the 1-year follow-up. Their SF-36 results after 1 year were equivalent to those observed in the no-touch group in our study, except for physical functioning, which was better in the study by Cohen et al., and mental health/emotional well-being, which was better in our study. This comparison between the PCI of a native vessel^[27]^ and the PCI of a no-touch vein graft suggests the hypothesis that no-touch SVGs are as suitable for PCI as a native coronary artery. A better HRQoL score thus correlates with the reduced intimal damage and subsequent atherosclerosis in the long term during the no-touch vein harvesting^[18]^.

### Limitations of the Study

The main limitation of the present study is the small number of individuals in the no-touch group (n=48), despite the high response rate (87.3%). However, the power was sufficient to detect significant differences between the two study groups on four of the eight RAND-36 scales. We also calculated effect sizes, which are independent of sample size, to estimate the magnitude of group differences. A second limitation is the retrospective study design, which means that HRQoL data before the operation were not available. Another possible limitation is the difference between the study groups in terms of follow-up time. Individuals treated with the no-touch technique had a shorter follow-up time than the conventionally treated patients (4.4 *vs.* 5.9 years, Table 1). According to Hokkanen et al.^[6]^, a main predictor of HRQoL after CABG is the initial age at CABG, with declining RAND-36 scores at the 12-year follow-up in patients older than 65 years at the time of the CABG. Our study groups showed comparable age at time of CABG, PCI, and HRQoL follow-up, indicating that age was not a confounding factor in the comparison between the two groups.

## CONCLUSION

At a mean follow-up of 5.4 years, patients who received PCI in no-touch SVGs showed significantly better HRQoL than those who received PCI in conventional vein grafts.

**Table:** 

Authors’ Roles & Responsibilities	
GF	Substantial contributions to the conception or design of the work; or the acquisition, analysis or interpretation of data for the work; drafting the work or revising it critically for important intellectual content; final approval of the version to be published
JK	Substantial contributions to the conception or design of the work; or the acquisition, analysis or interpretation of data for the work; drafting the work or revising it critically for important intellectual content; final approval of the version to be published
YC	Substantial contributions to the conception or design of the work; or the acquisition, analysis or interpretation of data for the work; final approval of the version to be published
HG	Substantial contributions to the conception or design of the work; or the acquisition, analysis or interpretation of data for the work; final approval of the version to be published
DS	Substantial contributions to the conception or design of the work; or the acquisition, analysis or interpretation of data for the work; final approval of the version to be published
NS	Substantial contributions to the conception or design of the work; or the acquisition, analysis or interpretation of data for the work; final approval of the version to be published

## References

[1] Noyez L, de Jager MJ, Markou AL. (2011). Quality of life after cardiac surgery: underresearched
research. Interact Cardiovasc Thorac Surg.

[2] Coelho PNMP, Miranda LMRPC, Barros PMP, Fragata JIG. (2019). Quality of life after elective cardiac surgery in elderly
patients. Interact Cardiovasc Thorac Surg.

[3] Fox NL, Hoogwerf BJ, Czajkowski S, Lindquist R, Dupuis G, Herd JA (2004). Quality of life after coronary artery bypass graft: results from
the POST CABG trial. Chest.

[4] Gjeilo KH, Stenseth R, Wahba A, Lydersen S, Klepstad P. (2018). Long-term health-related quality of life and survival after
cardiac surgery: a prospective study. J Thorac Cardiovasc Surg.

[5] Grand N, Bouchet JB, Zufferey P, Beraud AM, Awad S, Sandri F (2018). Quality of life after cardiac operations based on the minimal
clinically important difference concept. Ann Thorac Surg.

[6] Hokkanen M, Järvinen O, Huhtala H, Tarkka MR. A (2014). 12-year follow-up on the changes in health-related quality of
life after coronary artery bypass graft surgery. Eur J Cardiothorac Surg.

[7] Järvinen O, Hokkanen M, Huhtala H. (2019). Diabetics have inferior long-term survival and quality of life
after CABG. Int J Angiol.

[8] Pačarić S, Turk T, Erić I, Orkić Ž, Petek Erić A, Milostić-Srb A (2020). Assessment of the quality of life in patients before and after
coronary artery bypass grafting (CABG): a prospective study. Int J Environ Res Public Health.

[9] Peric V, Stolic R, Jovanovic A, Grbic R, Lazic B, Sovtic S (2017). Predictors of quality of life improvement after 2 years of
coronary artery bypass surgery. Ann Thorac Cardiovasc Surg.

[10] Perrotti A, Ecarnot F, Monaco F, Dorigo E, Monteleone P, Besch G (2019). Quality of life 10 years after cardiac surgery in adults: a
long-term follow-up study. Health Qual Life Outcomes.

[11] Samano N, Bodin L, Karlsson J, Geijer H, Arbeus M, Souza D. (2017). Graft patency is associated with higher health-related quality of
life after coronary artery bypass surgery. Interact Cardiovasc Thorac Surg.

[12] Brilakis ES, O'Donnell CI, Penny W, Armstrong EJ, Tsai T, Maddox TM (2016). Percutaneous coronary intervention in native coronary arteries
versus bypass grafts in patients with prior coronary artery bypass graft
surgery: insights from the veterans affairs clinical assessment, reporting,
and tracking program. JACC Cardiovasc Interv.

[13] Ferrari G, Geijer H, Cao Y, Souza D, Samano N. (2021). Percutaneous coronary intervention in saphenous vein grafts after
coronary artery bypass grafting: a systematic review and
meta-analysis. Scand Cardiovasc J.

[14] Souza D. (1996). A new no-touch preparation technique. Technical
notes. Scand J Thorac Cardiovasc Surg.

[15] Ramos De Souza D, Dashwood MR, Samano N (2017). Saphenous vein graft harvesting and patency: no-touch harvesting
is the answer. J Thorac Cardiovasc Surg.

[16] Samano N, Dashwood M, Souza D. (2018). No-touch vein grafts and the destiny of venous revascularization
in coronary artery bypass grafting-a 25th anniversary
perspective. Ann Cardiothorac Surg.

[17] Samano N, Pinheiro BB, Souza D. (2019). Surgical aspects of no-touch saphenous vein graft harvesting in
CABG: clinical and angiographic follow-up at 3 months. Braz J Cardiovasc Surg.

[18] Johansson BL, Souza DS, Bodin L, Filbey D, Loesch A, Geijer H (2010). Slower progression of atherosclerosis in vein grafts harvested
with 'no touch' technique compared with conventional harvesting technique in
coronary artery bypass grafting: an angiographic and intravascular
ultrasound study. Eur J Cardiothorac Surg.

[19] Pinheiro BB, Dashwood M, Souza DSR. (2021). The "no-touch" harvesting technique revives the position of the
saphenous vein as an important conduit in CABG surgery: 30-year
anniversary. Braz J Cardiovasc Surg.

[20] Samano N, Souza D, Pinheiro BB, Kopjar T, Dashwood M. (2020). Twenty-five years of no-touch saphenous vein harvesting for
coronary artery bypass grafting: structural observations and impact on graft
performance. Braz J Cardiovasc Surg.

[21] Brilakis ES, Rao SV, Banerjee S, Goldman S, Shunk KA, Holmes DR (2011). Percutaneous coronary intervention in native arteries versus
bypass grafts in prior coronary artery bypass grafting patients: a report
from the national cardiovascular data registry. JACC Cardiovasc Interv.

[22] Lichtenwalter C, de Lemos JA, Roesle M, Obel O, Holper EM, Haagen D (2009). Clinical presentation and angiographic characteristics of
saphenous vein graft failure after stenting: insights from the SOS (stenting
of saphenous vein grafts) trial. JACC Cardiovasc Interv.

[23] Bjorner JB, Wallenstein GV, Martin MC, Lin P, Blaisdell-Gross B, Tak Piech C (2007). Interpreting score differences in the SF-36 vitality scale: using
clinical conditions and functional outcomes to define the minimally
important difference. Curr Med Res Opin.

[24] Lahoud R, Chongthammakun V, Wu Y, Hawwa N, Brennan DM, Cho L. (2017). Comparing SF-36® scores versus biomarkers to predict
mortality in primary cardiac prevention patients. Eur J Intern Med.

[25] Järvinen O, Saarinen T, Julkunen J, Huhtala H, Tarkka MR. (2003). Changes in health-related quality of life and functional capacity
following coronary artery bypass graft surgery. Eur J Cardiothorac Surg.

[26] Samano N, Geijer H, Liden M, Fremes S, Bodin L, Souza D. (2015). The no-touch saphenous vein for coronary artery bypass grafting
maintains a patency, after 16 years, comparable to the left internal
thoracic artery: a randomized trial. J Thorac Cardiovasc Surg.

[27] Cohen DJ, Van Hout B, Serruys PW, Mohr FW, Macaya C, den Heijer P (2011). Quality of life after PCI with drug-eluting stents or
coronary-artery bypass surgery. N Engl J Med.

[28] Günal A, Aengevaeren WR, Gehlmann HR, Luijten JE, Bos JS, Verheugt FW. (2008). Outcome and quality of life one year after percutaneous coronary
interventions in octogenarians. Neth Heart J.

